# Secondary endolymphatic hydrops: a clinical and literature overview

**DOI:** 10.3389/fneur.2024.1525954

**Published:** 2025-02-05

**Authors:** Aïna Venkatasamy, Anne R. J. Péporté

**Affiliations:** ^1^Department of Radiology, IHU Strasbourg, Strasbourg, France; ^2^Plateforme Imageries du Vivant, Université de Paris, PARCC, INSERM, Paris, France; ^3^Department of Radiology Medical Physics, Faculty of Medicine, Medical Center-University of Freiburg, University of Freiburg, Freiburg, Germany; ^4^Equipe MLMS, Laboratoire ICUBE UMR7357, Strasbourg, France; ^5^Department of Radiology, Cantonal Hospital Frauenfeld, Frauenfeld, Switzerland

**Keywords:** hydropic ear disease, MRI, endolymphatic hydrops, secondary endolymphatic hydrops, inner ear disease, Menière disease, computed tomograghy, inner ear imaging

## Abstract

**Introduction and importance:**

Secondary endolymphatic hydrops (SEH) is a pathologic condition of the inner ear that usually manifests as episodic vertigo and fluctuating hearing loss, overlapping with other temporal bone pathologies and inner ear diseases.

**Methods:**

We searched Pubmed and the Cochrane database for English-language studies published through July 2024.

**Results:**

Fifty-four relevant studies and reviews were included in this review on secondary endolymphatic hydrops. This review presents a range of the underlying pathologies in endolymphatic hydrops, along with their corresponding radiological findings, while discussing the associated pathophysiological mechanisms. Secondary endolymphatic hydrops may result from cerebellopontine angle tumors, longstanding inner ear conditions, inner ear malformations, intracranial hypotension and recent investigations have highlighted the role of trauma and inflammation as key factors in SEH development.

**Discussion:**

Despite the diverse etiologies of SEH, the findings suggest that many of these conditions share a common final pathway in disrupting endolymphatic fluid balance. This review provides a better understanding of the pathophysiology and etiologies of this intricate disease process, thereby facilitating the diagnosis and treatment of the affected patients.

## 1 Introduction

The inner ear’s vestibular and cochlear components are critical for hearing and balance, with the maintenance of endolymphatic fluid homeostasis being essential for their proper function. Disruption in the delicate balance of endolymph and perilymph can lead to various pathological conditions, one of which is endolymphatic hydrops. Endolymphatic hydrops is characterized by an abnormal accumulation of endolymph within the membranous labyrinth, leading to a spectrum of clinical symptoms including vertigo, hearing loss, tinnitus, and aural fullness. These symptoms are often debilitating and can significantly impact a patient’s quality of life.

Traditionally, the diagnosis of endolymphatic hydrops was clinical, relying on symptomatology and audiometric findings, as direct visualization of the hydrops was not possible with earlier imaging techniques. However, advancements in magnetic resonance imaging (MRI) over the past decade have revolutionized our ability to diagnose and study this condition. The development of delayed post-gadolinium MRI techniques has allowed for the visualization of the endolymphatic and perilymphatic spaces, providing a direct assessment of endolymphatic hydrops (1). This breakthrough has not only improved the diagnostic accuracy for conditions like Meniere’s disease but has also led to the identification of secondary endolymphatic hydrops in a range of other otologic conditions (2).

In primary hydropic ear disease, no underlying cause can be identified on imaging. It is commonly referred to as Meniere’s disease (MD) and includes typical MD, its variants and/or atypical presentations (2). The endolymphatic sac epithelium is a salt-sensitive tissue similar to the kidney’s salt-absorbing epithelium, crucial for maintaining sodium and volume balance in the inner ear. The loss or absence of the endolymphatic sac epithelium has been proposed as the underlying cause of idiopathic EH and a key factor in the development of MD symptoms (3). On the other hand, in secondary hydropic ear disease, EH is observed in conjunction with various other otological disorders, including vestibular schwannoma (VS), inner ear malformations such as large vestibular aqueduct syndrome (LVAS), infections (e.g., labyrinthitis or meningitis), trauma, and malformations (2). In secondary hydropic ear disease, imaging plays a crucial role in differentiating these conditions, as overlapping symptoms can sometimes lead to their misattribution to another inner ear pathology.

In this review, we aim to provide a comprehensive analysis of secondary endolymphatic hydrops in non-hydropic temporal bone diseases. We will explore the underlying pathophysiology, the imaging characteristics of various conditions associated with secondary hydrops, and the clinical implications of these findings. By summarizing the latest research and clinical advancements, we seek to offer a clear overview on secondary endolymphatic hydrops (SEH) that enhances diagnostic accuracy and informs treatment strategies for affected patients.

## 2 Methods

We searched Pubmed and the Cochrane databases for English-language studies published through July 1st, 2024, for randomized clinical trials, systematic reviews, observational studies and relevant case reports. We also manually searched the references of selected articles, reviews and guidelines. All 54 selected articles were mutually agreed upon by the authors. Emphasis was given to provide information of interest to a general medical readership, while providing radiological findings and discussing the associated pathophysiological mechanisms.

### 2.1 Imaging presentation

Traditionally, MRI has been the cornerstone for ruling out differential diagnoses like vestibular schwannoma or other cerebellopontine angle pathologies, as well as some inner ear malformations. However, over the past decade, MRI techniques have evolved to visualize the morphologic features of endolymphatic hydrops (EH), particularly utilizing high-resolution imaging to assess temporal bone diseases, with high sensitivity (69–92%) and specificity (78–96%) (4, 5). Non-Contrast MRI Techniques, such as heavily T2-weighted sequence (Constructive Interference in Steady State or Fast Imaging Employing Steady-State Acquisition), sensitive to fluid changes, allow the assessment of endolymphatic and perilymphatic compartments, especially in temporal bone diseases causing changes in the protein content of inner ear fluids, such as vestibular schwannomas (6–11). In a similar way to T2w GRE (gradient-echo), fluid-attenuated inversion recovery (FLAIR) sequences, have proven to be valuable in revealing these perilymphatic protein alterations. In recent years, contrast-enhanced MRI emerged as the gold standard technique for evaluating secondary EH, particularly in patients with non-hydropic temporal bone diseases. A standard protocol involves using a 3D-FLAIR or 3D inversion recovery (IR) sequence, performed 24 h post-intratympanic gadolinium injection (off-label use) or 2.5 to 4 h after intravenous gadolinium administration (4). The latter method is most common in clinical practice. The contrast material diffuses into the perilymph, while the endolymph remains non-enhancing, allowing clear delineation between these two compartments and identifying areas of abnormal hydrops. MRI-based classification systems for EH offer valuable tools for clinicians to objectively evaluate the extent and progression of EH in the context of both primary and secondary cause (4, 11–13). Continued advancements in MRI technology, particularly in 3D imaging and contrast techniques, hold promise for improving diagnostic accuracy and treatment planning for patients with secondary EH.

### 2.2 Main causes of secondary endolymphatic hydrops

Secondary endolymphatic hydrops has several etiologies, summarized in Table 1.

**Table 1:** Main causes of secondary endolymphatic hydrops.

Cerebello-pontine angle tumors	Vestibular schwannomas
	Internal auditory canal meningioma
	Endolymphatic sac tumor
Inner ear malformations	Pathologic third window lesions Superior semicircular canal dehiscence syndrome (SSCD) Large vestibular aqueduct syndrome (LVAS)
	Lateral canal vestibular dysplasia (LCVD)
Trauma to the inner ear	Surgical procedures
	Radiation therapy
	Noise-induced trauma
	Blunt-force trauma
Infections	Otitis media, labyrinthitis, meningitis
Longstanding inner ear diseases	Otosclerosis
Spontaneous intracranial hypotension	

Etiologies of secondary endolymphatic hydrops.

#### 2.2.1 Tumor-related secondary endolymphatic hydrops

##### 2.2.1.1 Vestibular and primary inner ear schwannomas

Vestibular schwannomas (VS) are the most frequent tumors of the cerebellopontine angle (CPA), presenting with unilateral hearing loss, tinnitus, and/or balance issues, overlapping symptoms of endolymphatic hydrops, thus complicating the clinical diagnosis. On MRI, VS demonstrate changes in signal intensity within the perilymph on MRI on sequences sensitive to protein variations (e.g., T2w GRE or FLAIR sequences) (14–16). The pathophysiological explanation for these signal changes, is that the tumor may induce biochemical alterations of the composition of the perilymph, through the secretion of several proteins (6, 17–20). For instance, Rasmussen et al. identified 314 proteins in total, 91 of which were detected in 12 out of 15 patients. In particular, the alpha-2-HS-glycoprotein (P02765, found in the extra-cellular region), was observed in all their patients and appeared to be an independent variable for tumor-associated hearing loss (18). Tumor-related changes in protein content of the perilymph may also indirectly affect the blood-perilymph barrier integrity (18, 21). All these osmotic alterations can lead to pressure changes in the perilymphatic and endolymphatic compartments of the inner ear, contributing to the occurrence of SEH (2).

**Figure 1:**
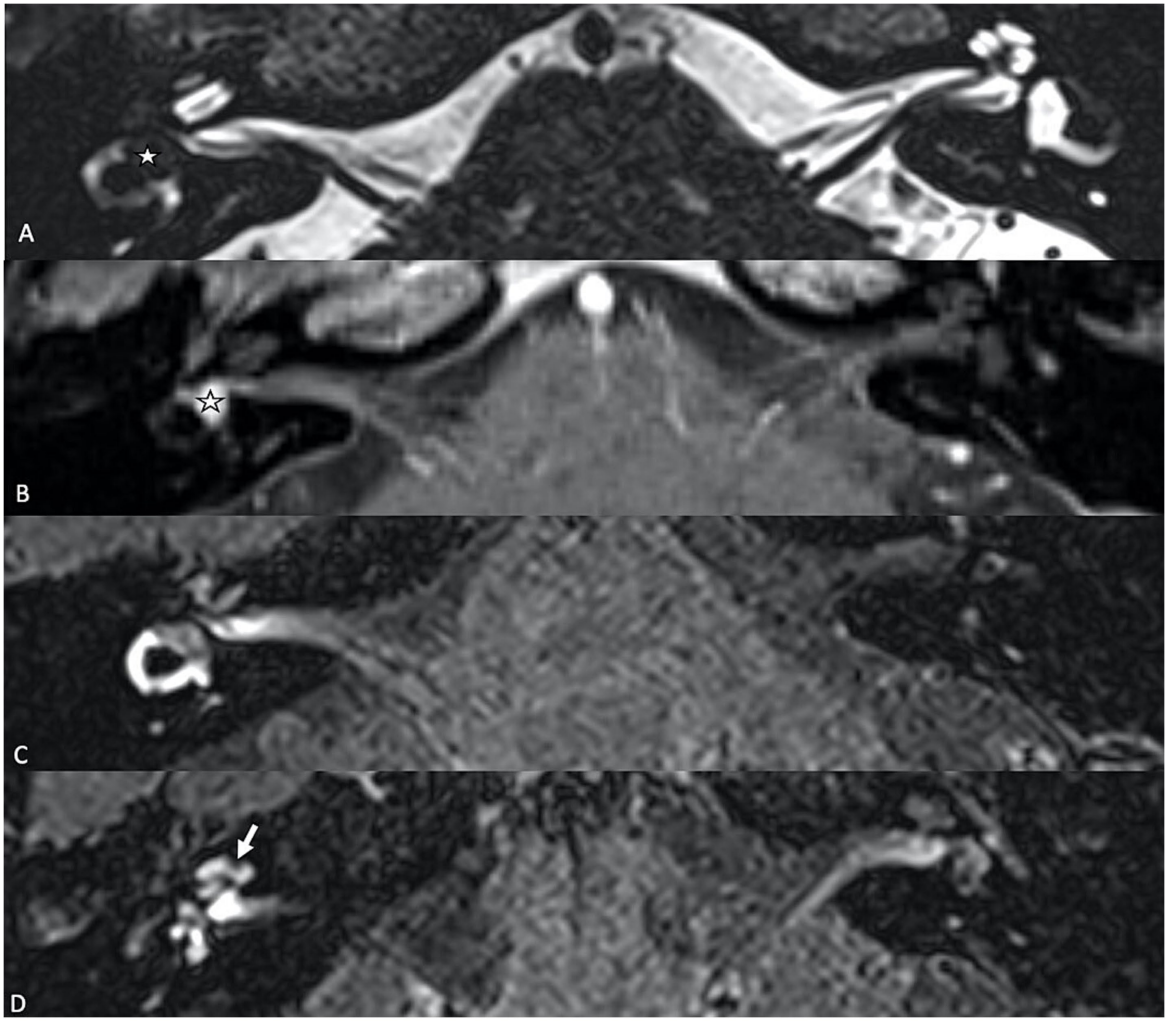
**(A)** T2-weighted axial MR image showing a primary inner ear (intralabyrinthine) schwannoma (star) in the right vestibular cistern. **(B)** On the T1-weighted contrast-enhanced image, the tumor appears as a homogenously enhancing nodule (star). **(C,D)** Delayed 4 h30 FLAIR images showing distension of the right cochlear duct consistent with cochlear endolymphatic hydrops (arrow).

In patients with VS, SEH is observed in approximately 20–30% of cases (14–16). Notably, secondary EH affects the contralateral ear in about 15% of these cases, suggesting a potential systemic component (14). Primary inner ear schwannomas, also known as intra-labyrinthine schwannomas, are much rarer than typical vestibular schwannomas and are regarded as a distinct clinical entity, because they originate from within the labyrinth itself (22). SEH is notably more prevalent in cases of primary inner ear schwannoma (Figure 1), occurring in 47–67% of such instances. In these cases, the proximity of the tumor to the peri-and endo-lymphatic spaces may facilitate the diffusion of tumor-secreted proteins, potentially exacerbating the hydrops (23, 24).

##### 2.2.1.2 Internal auditory canal meningioma

**Figure 2:**
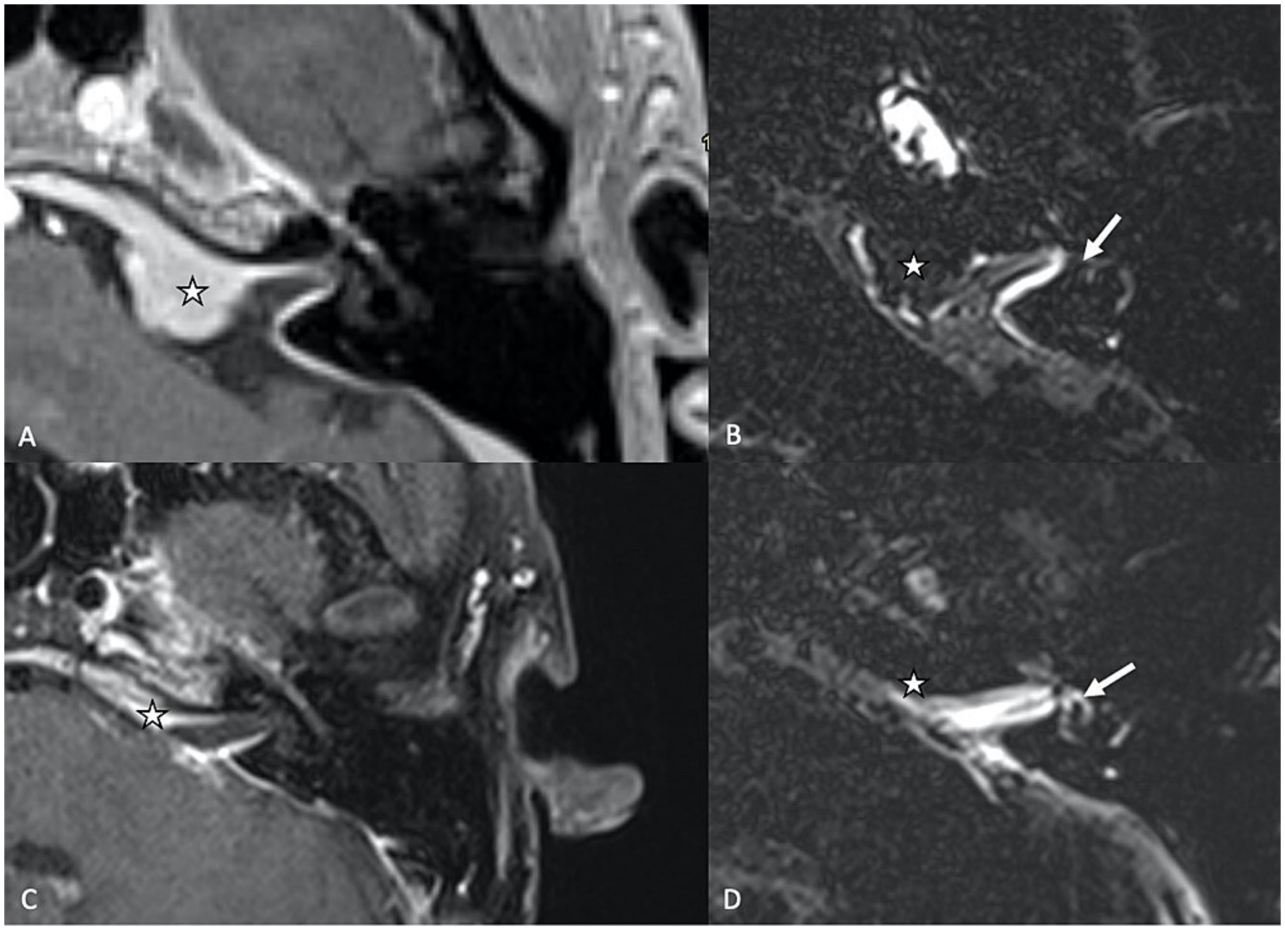
**(A)** Axial T1-weighed contrast-enhanced MR sequence showing a cerebello-pontine angle meningioma (star) extending into the internal auditory canal. **(B)** Delayed (4 h30) FLAIR image showing vestibular endolymphatic hydrops (grade 3) with full obliteration of the bony vestibule. **(C)** T1-weighed contrast-enhanced sequence showing residual scar tissue (star) tissue after surgical resection. **(D)** On the delayed FLAIR sequence, there is regression of the endolymphatic space dilation, which coincides with the clinical resolution of vertigo attacks for the patient.

Meningiomas are slow-growing, benign tumors that typically arise from the meninges, and when they extend into the internal auditory canal (Figure 2), that can compress or displace critical inner ear structures. Patients may present with progressive hearing loss, tinnitus, vertigo, or balance disturbances, which overlap with those seen in SEH. Perilymphatic signal changes have also been described in cerebellopontine meningiomas extending into the internal auditory canal on T2W GE sequence, serving as an additional diagnostic feature to differentiate between the two tumors (6). In rare cases, posterior fossa meningiomas may affect the endolymphatic sac, which may lead to endolymphatic hydrops, due to mechanical obstructions like other posterior fossa neoplasms (25, 26).

##### 2.2.1.3 Endolymphatic sac tumor

Endolymphatic sac tumors (ELST) are rare, hyper-vascular locally aggressive papillary neoplasm that arise in the retro-labyrinthine region of the temporal bone (27). The majority of ELSTs occur sporadically, but approximately 30% of cases are associated with Von Hippel–Lindau’s disease (28). The clinical presentation of ELSTs is predominantly auditory with symptoms such as persistent sensorineural hearing loss (SHNL; sudden in nearly 50% of cases), along with tinnitus and vertigo. The SHNL in ELST is thought to result from invasion of the otic capsule, tumor-associated intralabyrinthine hemorrhage, or the development of endolymphatic hydrops on the side of the tumor (29). The contralateral side usually does not exhibit signs of hydrops (30).

#### 2.2.2 Inner ear malformations

##### 2.2.2.1 Pathologic third window lesions

**Figure 3:**
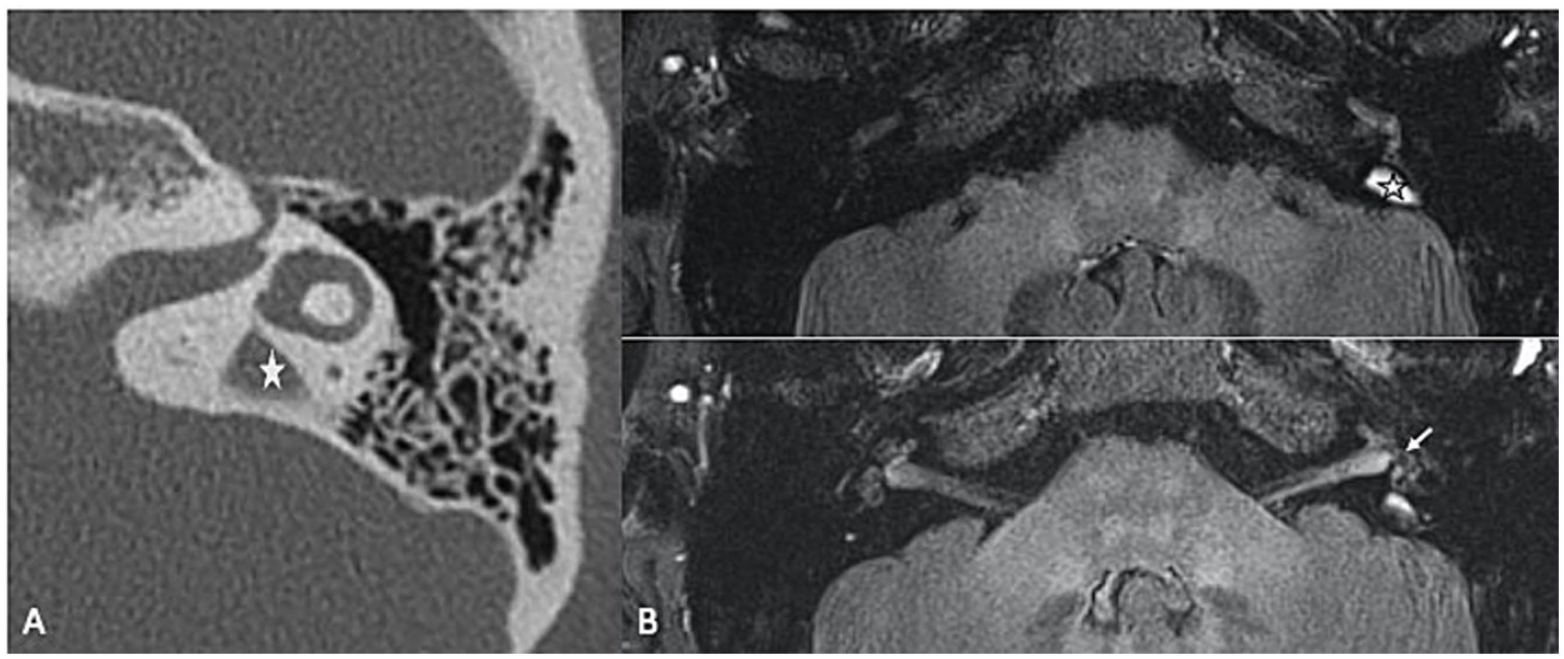
**(A)** High resolution CT of the temporal bone showing an enlarged vestibular aqueduct on the left (LVAS) (star). **(B)** 3D TSE FLAIR sequence showing a LVAS (star) together with signs of SHE (enlarged saccule, arrow).

Inner ear malformations, particularly those resulting in “pathologic third window lesions” like superior semicircular canal dehiscence syndrome (SSCD) and large vestibular aqueduct syndrome (LVAS), are known to disrupt the delicate fluid balance within the inner ear, altering perilymphatic pressure dynamics, thus contributing to the development of SEH. The mechanism behind this phenomenon may involve shifts in protein concentration that affect osmotic pressure between the endolymphatic and perilymphatic compartments, or direct pressure changes within the inner ear due to the presence of a third window. In SSCD, a dehiscence in the superior semicircular canal creates an abnormal third window, alongside the oval and round windows, through which sound and pressure stimuli can abnormally move the endolymph (Tullio phenomenon, with vertigo and nystagmus triggered by loud sounds). Secondary EH is a common finding in SSCD, with reported prevalence rates ranging from 27.3 to 80% of the cases (31, 32). Large vestibular aqueduct syndrome (LVAS) is a congenital condition characterized by an abnormally enlarged vestibular aqueduct (Figure 3), also associated with SEH. It is a recognized cause of unilateral or bilateral progressive sensorineural hearing loss among the pediatric and adolescent population. The exact etiology of hearing loss in patients with LVAS is still unknown. One significant hypothesis is that the hyperosmolar, protein-rich fluid present in the enlarged endolymphatic sac refluxes into the endolymphatic spaces of the cochlea, thereby damaging the hair cells, leading to SEH (31, 33).

##### 2.2.2.2 Lateral canal vestibular dysplasia (LCVD)

**Figure 4:**
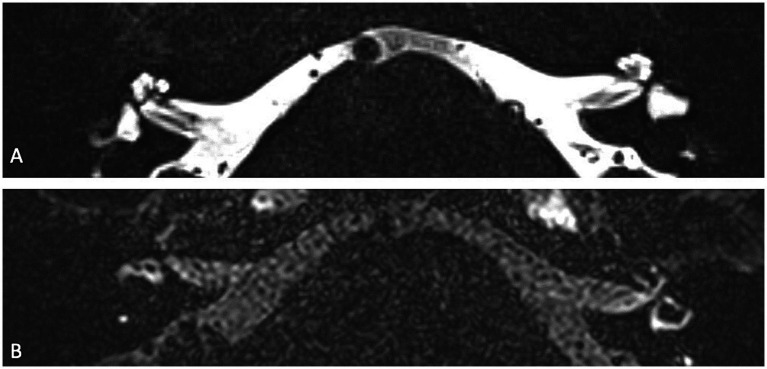
Axial 3D T2w TSE **(A)** and axial 4 h delayed post-Gd 3D Space FLAIR **(B)** showing a left-sided cystic dilatation of the saccule and utricle, that are fused on a congenital basis. On the delayed FLAIR image, there is a grade 2 vestibular hydrops (confluence of the saccule and utricle surrounded by a thin perilymphatic enhancement rim).

Lateral canal vestibular dysplasia (LCVD) is a common congenital malformation of the inner ear, associated with vestibular dysfunction and varying degrees of hearing impairment, that primarily affects the lateral semicircular canal, an essential structure responsible for sensing horizontal head movements. The severity of dysplasia may vary from a short and broad lateral semicircular canal to a single fluid-filled cavity (Figure 4) confluent with the vestibule (34). Naganawa et al. demonstrated that patients with LCVD had a significantly increased vestibular endolymphatic ratio compared to controls, suggesting that the malformation affects endolymphatic fluid regulation within the vestibular system. Similarly, a strong negative correlation was found between the central bony island area and the size of the endolymph. The authors hypothesized that this increase in vestibular endolymphatic space is a result of the dysplasia impairing the normal clearance or absorption of endolymph, further contributing to the development of SEH (35). However, Niu et al. did not observe significant vestibular hydrops in patients with semi-circular malformations (36). Yun et al. expanded upon this, demonstrating that vestibular dysfunction in LCVD is not limited to the lateral canal, nor to hydrops alone, but can affect the broader vestibular system, leading to more complex vestibulocochlear symptoms (34). Thus, the observation of SEH in LCVD highlights the complex relationship between congenital malformations and secondary hydrops, where abnormal inner ear anatomy predisposes patients to fluid dynamic disturbances.

#### 2.2.3 Trauma to the inner ear (blunt force trauma, surgery, noise-induced trauma or radiation)

Trauma to the inner ear, whether from blunt force injuries, surgical interventions, radiation or noise-induced damage, which may disrupt the delicate fluid balance within the inner ear, can potentially lead to SEH (37).

##### 2.2.3.1 Surgical procedures

Surgical procedures may contribute to SHE in some cases. For instance, cochlear implantation, especially when associated with cochleostomy, has been associated with SEH in a portion of patients, with reports suggesting an incidence of 42–59% (38). This suggests that postoperative disruption of the normal inner ear balance could influence inner ear fluid dynamics, resulting in SEH. Several potential pathophysiological explanations have been suggested including a blockage of the endolymphatic system or the occurrence of cochlear inflammation (39). Furthermore, animal model studies in guinea pigs have shown that endolymphatic sac ablation can lead to mild hydrops within days post-surgery (40). This hydrops initially affects the saccule, endolymphatic sinus, and Reissner’s membrane, with progression to severe distension of Reissner’s membrane into the scala vestibuli, occurring within months (41).

##### 2.2.3.2 Radiation therapy

Post-irradiation SEH is a potential complication following radiation treatment of the head and neck region, leading to disturbances in inner ear fluid homeostasis. Radiation-induced damage to the cochlear and vestibular structures can disrupt the delicate balance between perilymphatic and endolymphatic pressures, contributing to the onset of SEH. This fluid imbalance may manifest as hearing loss, vertigo, or tinnitus, with the severity and presentation of symptoms varying depending on the extent of inner ear damage. Additionally, post-irradiation SEH should be distinguished from other radiation-induced auditory conditions, such as sudden SNHL (42). While both conditions may arise following radiation therapy, they differ in their underlying pathophysiology and clinical course. SEH is typically associated with a more gradual onset of symptoms, often accompanied by episodic vertigo, whereas sudden SNHL tends to present suddenly without vestibular involvement (2). This underscores the importance of considering SEH as a potential diagnosis in patients presenting with auditory or vestibular symptoms post-radiation and highlights the utility of MRI for detecting SEH in these cases (42).

##### 2.2.3.3 Noise-induced trauma

Noise-induced trauma may also contribute to the development of SEH. Acoustic trauma, such as high-intensity sound exposure, can generate pressure-striking forces on the membranous labyrinth, and disrupt endolymphatic fluid balance, potentially leading to SEH. For instance, mice exposed to 100 dB sound pressure level noise for two hours, developed EH and had reduced synaptic counts in the basal and middle regions of the cochlea and similar results were observed on guinea pigs (43, 44).

##### 2.2.3.4 Blunt-force trauma

Blunt-force trauma (i.e., mechanical trauma to the inner ear), may also result in SEH. Physical impacts from blunt injuries can potentially disrupt the intricate structures of the inner ear, or cause pressure changes, both leading to changes in fluid dynamics, thus contributing to SEH (37). The severity and presentation of hydrops might vary depending on the extent of the trauma and its impact on the inner ear’s fluid compartments. Traumatic EH involves the accumulation of endolymph in the cochlear duct caused by traumatic insult. Possible mechanisms include: (1) a disruption in the normal perilymph-endolymph pressure relationship due to bony labyrinth fistulization; (2) direct trauma to the membranous labyrinth, potentially leading to fluid collection in the cochlear duct that may resolve over time; and (3) injury to the endolymphatic fluid drainage pathway, including temporal bone fractures involving the vestibule or the vestibular aqueduct. This can cause fibro-osseous blockage of the endolymphatic duct and surgical injury to the saccule with obstruction of the longitudinal flow of endolymph, resulting in SEH that may be delayed in onset and usually persists. The diagnosis of traumatic endolymphatic hydrops generally involves a history of trauma (e.g., barotrauma, blow to the head…); typical symptoms of EH (e.g., fullness, tinnitus, fluctuant hearing loss, and/or episodic vertigo); and characteristic electrocochleography findings (45).

#### 2.2.4 Infections

**Figure 5:**
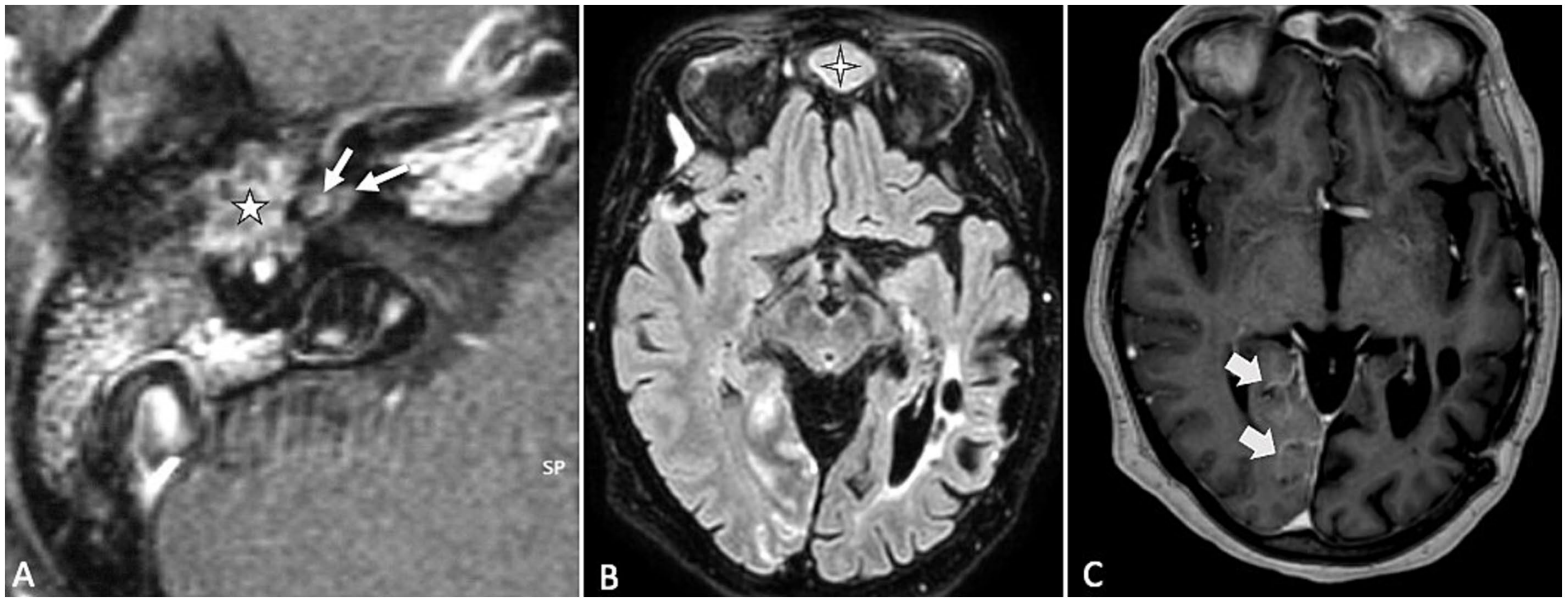
**(A)** Contrast-enhanced MRI, T1-weighted sequence showing acute otitis media with inflammatory enhancing granulation tissue filling of the right middle ear cavity (star) and suppurative labyrinthitis with non-nodular contrast enhancement (thin arrows) in the basal and second turns of the cochlea. **(B,C)** Another patient: FLAIR and T1-weighted sequences post-gadolinium injection reveal frontal sinusitis (cross symbol) and leptomeningeal enhancement (large arrow) in the right occipital region, consistent with occipital leptomeningitis.

Infections such as otitis media (Figure 5A), suppurative labyrinthitis or meningitis (Figures 5B,C) have been linked to the development of SEH. Kaya et al. observed that 36% of temporal bones affected by suppurative labyrinthitis exhibited SEH (*n* = 10/28), compared to only 5% in the control group (*n* = 1/20, *p* = 0.006) (46). This suggests that the inflammatory processes associated with these infections may disrupt the normal fluid balance within the inner ear, leading to the development of SEH. Additionally, Silverstein et al. also described elevated levels of proteins within the perilymph of patients with acute bacterial labyrinthitis, which could also contribute to osmotic imbalance between the perilymphatic and endolymphatic compartments, resulting in SEH (47). All these findings underscore the importance of considering SEH in the differential or co-occurring diagnosis of patients with a history of severe inner ear infections.

#### 2.2.5 Longstanding inner ear disease

##### 2.2.5.1 Otosclerosis

**Figure 6:**
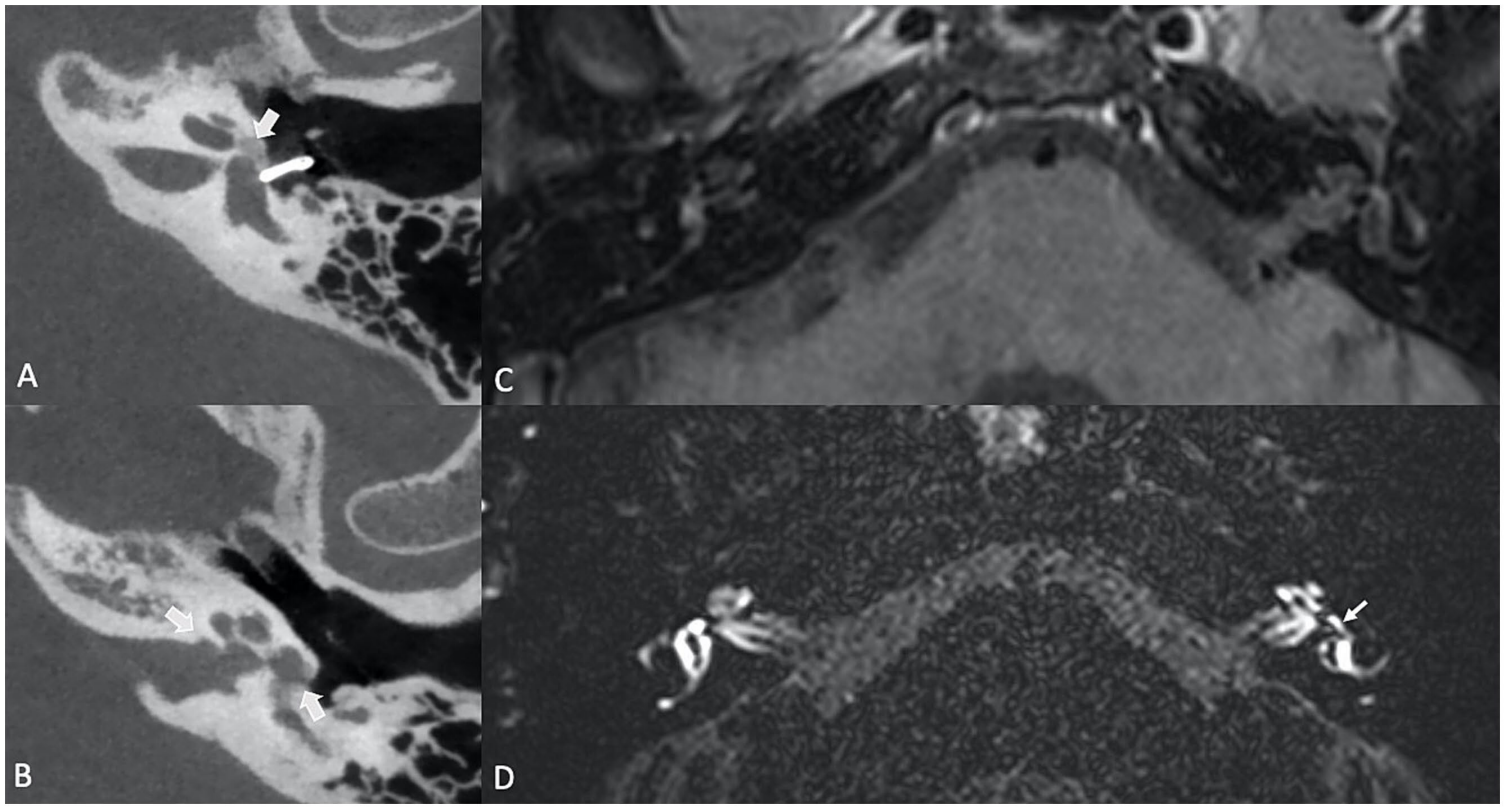
**(A,B)** Axial high resolution temporal bone Cone Beam CT (CBCT) in a patient with longstanding fenestral and retrofenestral otosclerosis, presenting with otospongiotic plaques at the fissula ante fenestram, the cochlea and the internal auditory canal (thick arrows) and a piston. **(C)** Contrast-enhanced, axial T1-weighted MR sequence and **(D)** Contrast-enhanced, delayed SPACE FLAIR MR sequence, showing a cochlear hydrops (grade 1) with irregular dilatation of the scala media, partial obstruction of the scala vestibuli and a vestibular hydrops grade 2 (small arrow) with a beginning of confluence of the enlarged saccule and utricle.

SEH has also been reported in longstanding inner ear conditions such as otosclerosis, with prevalence rates ranging from 26 to 55%, whether the otosclerosis has been surgically treated or not (48, 49). While the precise mechanism by which SEH develops in otosclerosis remains unclear, preoperative EH has been suggested as a potential risk factor for inner ear disturbances following stapes surgery (50). Thus, acute episodes of sensorineural hearing loss or vertigo in patients with otosclerosis may be indicative of underlying SEH, though the condition does not always present with noticeable clinical manifestations. Thus, clinicians should maintain a high index of suspicion, especially when patients experience sudden changes in symptoms (Figure 6), as this may warrant an MRI evaluation to confirm the presence of SEH (51).

##### 2.2.5.2 Spontaneous intracranial hypotension

**Figure 7:**
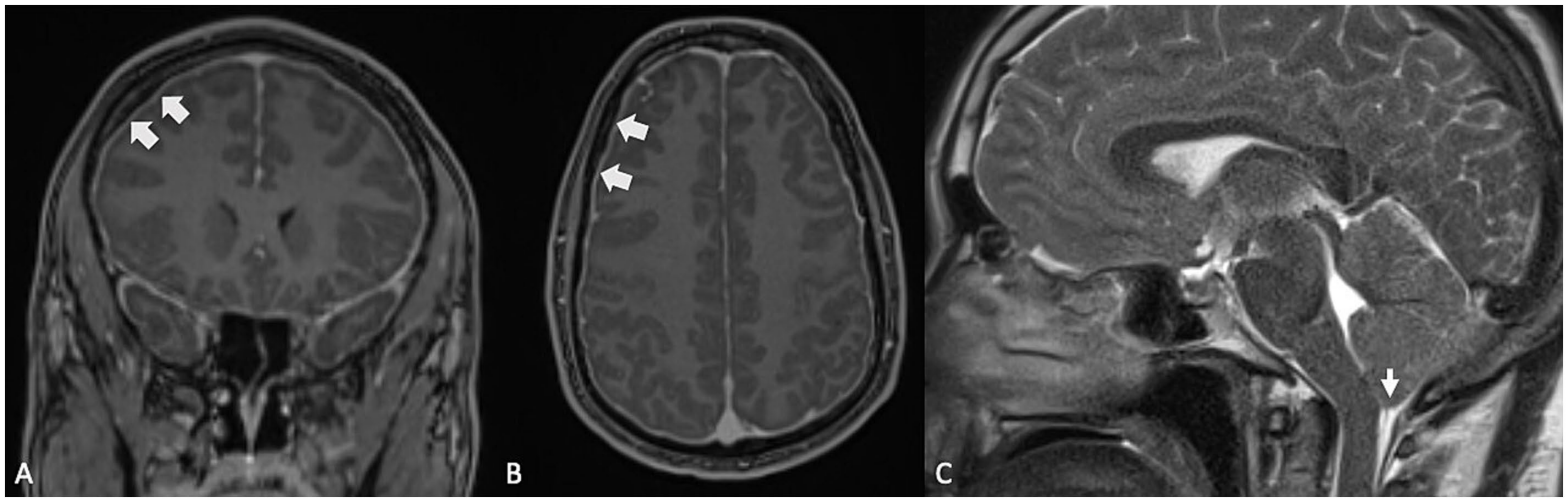
**(A,B)** Contrast-enhanced MRI, T1-weighted sequences in coronal and axial planes, showing diffuse pachymeningeal enhancement and thickening (large arrows), indicative of intracranial hypotension. **(C)** Same patient: T2-weighted sequence, sagittal plane, demonstrating moderate sagging brainstem and cerebellar tonsillar descent, also consistent with intracranial hypotension.

Spontaneous intracranial hypotension (Figure 7), characterized by reduced cerebrospinal fluid pressure, has been linked to the development of SEH (52–54). This association is thought to stem from changes in the pressure gradient between the perilymph and endolymph within the inner ear. Perilymphatic fluid is believed to communicate with the CSF via the cochlear aqueduct, and a drop in CSF pressure results in a corresponding decrease in perilymphatic pressure. When perilymphatic pressure falls below the endolymphatic pressure, it can lead to the dilation of the endolymphatic space, contributing to SEH. The underlying mechanism is hypothesized as a “vacuum phenomenon,” where negative pressure within the perilymphatic compartment causes an expansion of the endolymphatic space, promoting hydrops, highlighting the delicate balance of fluid dynamics in the inner ear and how intracranial pressure changes can influence inner ear function.

## 3 Discussion

Radiological, clinical, and histopathological studies demonstrate that endolymphatic hydrops (EH) can arise from various inner ear pathologies. In cerebellopontine angle tumors (e.g., vestibular schwannomas, meningiomas, and endolymphatic sac tumors) mechanisms like increased protein concentration in the perilymphatic space, invasion of the otic capsule, or dilation of the membranous labyrinth contribute to the development of EH. In cases of inner ear malformations, such as malformations with a “third window” effect (e.g., superior semicircular canal dehiscence, large vestibular aqueduct syndrome) or lateral canal vestibular dysplasia, the abnormal anatomy alters the pressure dynamics within the inner ear, disrupting fluid regulation and leading to secondary EH. Similarly, trauma-related causes, including blunt force trauma, noise exposure, radiation, and surgical interventions, result in fluid imbalance and/or cochleovestibular inflammation, further contributing to secondary hydrops. A comparable mechanism is seen in SEH due to intracranial hypotension, where a pressure gradient between the perilymph and endolymph triggers endolymphatic space dilation. Examining the diverse conditions that contribute to SEH, a consistent pattern or common final pathway emerges in the underlying mechanisms. This pathway involves two primary factors: (1) alterations in inner ear fluid dynamics due to pressure changes, and (2) disturbances in protein concentrations within the inner ear. These factors collectively disrupt the delicate balance of the endolymphatic system, leading to the development of secondary hydrops. Understanding this dual mechanism of pressure shifts and protein alterations provides insight into the shared pathophysiological process across various etiologies of secondary endolymphatic hydrops.

A key limitation of the current body of research is the relatively small sample size in many of the studies reviewed, most of which are retrospective in nature. This limits the generalizability of the findings and the strength of the conclusions regarding the pathophysiology of SEH. To establish a more robust understanding, larger, prospective studies, and potentially experimental investigations, are needed to confirm the proposed mechanisms and their associated imaging findings. These future studies would help solidify the relationship between secondary endolymphatic hydrops and its diverse etiologies.

## 4 Conclusion

A wide range of inner ear pathologies can lead to secondary endolymphatic hydrops (SEH), often presenting with overlapping symptoms that make distinguishing between different conditions challenging. This review offers a comprehensive overview of SEH, highlighting the diverse causes and mechanisms behind its development. By integrating detailed case illustrations and radiological insights, this work aims to enhance diagnostic accuracy and improve clinical management. A better understanding of these conditions will aid clinicians in early detection, allowing for more targeted interventions and improved patient outcome.
